# Incidence of Colorectal Cancer in Patients Diagnosed With Pyogenic Liver Abscess

**DOI:** 10.1001/jamanetworkopen.2023.48218

**Published:** 2023-12-18

**Authors:** Hiroyuki Suzuki, Ian Kidder, Tomohiro Tanaka, Michihiko Goto

**Affiliations:** 1 Center for Access & Delivery Research & Evaluation, Iowa City Veterans Affairs Health Care System, Iowa City, Iowa; 2 Division of Infectious Diseases, Department of Internal Medicine, University of Iowa, Iowa City; 3 Division of General Internal Medicine, Department of Internal Medicine, University of Iowa, Iowa City; 4 Division of Gastroenterology and Hepatology, Department of Internal Medicine, University of Iowa, Iowa City

## Abstract

**Importance:**

Colorectal cancer (CRC) can compromise the mucosal barrier and subsequently allow a route for bacterial invasion into the portal system or systemic circulation. Despite preliminary data suggesting that patients who experienced pyogenic liver abscess (PLA) have higher CRC incidence rates, data from outside the Southeast Asian population are sparse.

**Objective:**

To investigate whether there is an association between PLA and the subsequent incidence of diagnosed CRC using the Veterans Health Administration (VHA) cohort.

**Design, Setting, and Participants:**

A patient-level matched retrospective cohort study was conducted at 127 VHA hospitals across the US from January 1, 2003, to December 31, 2020. Patients were followed up to 10 years from PLA diagnosis. Data analysis was performed from April 14, 2002, to October 31, 2023. All patients who were admitted to VHA hospitals with a diagnosis of PLA were included. For each patient with PLA, up to 3 controls without diagnosis of PLA, matching age, sex, and health care facility, were selected.

**Exposure:**

Pyogenic liver abscess.

**Main Outcomes and Measures:**

The primary outcome was CRC diagnosis during the follow-up period. A multivariable Fine-Gray subdistribution regression model with time-dependent coefficient was used to estimate the time-varying hazard ratio (HR) of CRC incidence while accounting for mortality as a competing event.

**Results:**

A total of 8286 patients with PLA (male, 96.5%; mean [SD] age, 65.8 [11.9] years) and 23 201 patient-level matched controls (male, 96.3%; mean age, 65.3 [11.7] years) were included. A diagnosis of CRC was found in a significantly higher proportion of patients with PLA compared with controls (1.9% [159 of 8286] vs 0.8% [196 of 23 201]; *P* < .001). The incidence of CRC was significantly higher among patients with PLA during the first 3 years from PLA diagnosis (HR, 3.64; 95% CI, 2.70-4.91 at 0.5 years; HR, 2.51; 95% CI, 1.93-3.26 at 1 year; HR, 1.74; 95% CI, 1.33-2.28 at 2 years; and HR, 1.41; 95% CI, 1.05-1.89 at 3 years), but not significant after 3 years. This association was not observed among patients whose PLA was likely secondary to cholangitis or cholecystitis (HR, 1.78; 95% CI, 0.89-3.56 at 0.5 years).

**Conclusions and Relevance:**

In this patient-level matched retrospective cohort study, a significantly higher incidence of CRC was observed up to 3 years from PLA diagnosis. The findings suggest that offering CRC screening for patients with cryptogenic PLA may be useful, especially patients who have not been screened according to guidelines.

## Key Points

**Question:**

Is pyogenic liver abscess associated with the diagnosis of colorectal cancer?

**Findings:**

In this cohort study conducted over an 18-year period at 127 Veterans Affairs hospitals including 8286 patients, there was an association between a higher incidence of colorectal cancer rates within 3 years of pyogenic liver abscess diagnosis. The association was not observed if the cause of pyogenic liver abscess was considered secondary to cholangitis or cholecystitis.

**Meaning:**

The findings of this study suggest that colorectal cancer screening may be useful for patients with cryptogenic pyogenic liver abscess, especially in patients who have not been screened according to guidelines.

## Teaser

This cohort study examines the incidence of colorectal cancer in patients with a previous diagnosis of pyogenic liver disease.

## Introduction

Colorectal cancer (CRC) is a common and potentially life-threatening disease. It is the third most common cause of cancer death in both men and women, with an estimated 52 550 persons in the US projected to die from CRC in 2023.^1^ Most patients with early-stage CRC remain asymptomatic. Screening for asymptomatic individuals for CRC has been shown to detect asymptomatic early-stage cancer and improve mortality^2^ and therefore is advocated by major societies and preventive care organizations.^3,4,5,6^ While participation in CRC screening could be improved through organized screening programs,^7^ studies have found that 21.6% of eligible adults in the US had never been screened^8^ and 31.2% were not current with screening.^9^

Colorectal cancer can compromise the mucosal barrier and subsequently allow a route for bacterial invasion into the portal system or systemic circulation.^10^ Therefore, the occurrence of infection can be a first sign of silent CRC. Certain infectious diseases, such as *Streptococcus gallolyticus* bacteremia,^11^ anaerobic bacteremia,^12,13^ and pyogenic liver abscess (PLA), are reported to have an association with the incidence of CRC. A population-based retrospective cohort study conducted in Taiwan assessed the association between PLA and subsequent gastrointestinal cancers including CRC.^14^ In that study, patients were 5.5 times more likely to have CRC within 10 years from PLA diagnosis. More recently, a meta-analysis was conducted to assess the prevalence of CRC in patients with cryptogenic PLA.^15^ Among 12 articles identified, including the population-based study in Taiwan, the incidence rate of CRC in patients with PLA was 7-fold higher compared with patients without PLA, although the result might have been biased as all patients in the PLA group in the meta-analysis had received colonoscopy. Most of the studies included in that meta-analysis were conducted in Southeast Asia, and 1 small study was conducted in the US.^16^

Despite preliminary data suggesting that patients who experienced PLA have a higher CRC incidence, there are no professional guidelines to recommend CRC screening for patients who are diagnosed with PLA. In addition, epidemiologic studies about this association are mainly from Southeast Asia, with a few studies available from the US, where population risk factors for CRC and availability of CRC screening are greatly different from those in Southeast Asian countries.^17^ We investigated whether PLA is associated with the subsequent incidence of CRC using the Veterans Health Administration (VHA) cohort, which is the largest integrated health care system in the US.

## Methods

We conducted a patient-level, matched retrospective cohort study of all patients who were admitted to acute-care units at VHA hospitals with a PLA diagnosis (*International Classification of Diseases, 9th Revision* [*ICD-9*] code 572.0 or *International Statistical Classification of Diseases and Related Health Problems, 10th Revision* [*ICD-10*] code K75.0) from January 1, 2003, to December 31, 2020. Patients were identified from the VHA Corporate Data Warehouse using the Veterans Affairs Informatics and Computing Infrastructure. If a patient had more than 1 hospitalization, only the first hospitalization was used. Patients were excluded if CRC was diagnosed before PLA. We conducted manual medical record reviews of 100 randomly selected patients to verify the accuracy of the diagnostic codes. For each patient with PLA, we identified up to 3 control patients without a diagnosis of PLA who had established primary care within the VHA system when the case patient was diagnosed with PLA by matching age (±5 years), sex, and primary care facility. Control patients were enrolled in the follow-up cohort when the corresponding case patient was diagnosed with PLA. This study was approved by the institutional review boards of the University of Iowa and the Iowa City Veterans Affairs Health Care System Research and Development Committee. A waiver for informed consent was granted because this study only used deidentified data available in database. This study follows Strengthening the Reporting. of Observational Studies in Epidemiology (STROBE) reporting guideline.^18^

We obtained demographic characteristics, comorbidities, and previous CRC screening (colonoscopy or sigmoidoscopy conducted within the VHA system) for all patients with PLA and controls without a diagnosis of PLA from the Corporate Data Warehouse. Race was determined as White, Black and Other (including Asian, Native American, Pacific Islander, multirace, unknown, decline to answer, or missing) based on patient self-report. Tools used for measure included the Department of Veterans Affairs Alcohol Use Disorders Identification Test-C^19^ and the Centers for Disease Control and Prevention Social Vulnerability Index.^20^ For patients with PLA, we also obtained the presence or absence of concurrent biliary infection (cholecystitis or cholangitis) at the time of PLA diagnosis, predisposing conditions for biliary tract infections, such as biliary tract surgery, endoscopic retrograde cholangiopancreatography with sphincterotomy, tumor of the biliary tract or pancreatic head, cholelithiasis, liver transplant before PLA diagnosis, and cultures (blood or abscess) within 14 days from PLA diagnosis where available (eTable 1 in Supplement 1). We regarded PLA as likely to be secondary to biliary infection when the patient also had a diagnostic code of cholangitis or cholecystitis from 28 days before to 7 days after PLA diagnosis. Similarly, if a patient with PLA had a predisposing condition for biliary tract infection before PLA diagnosis, we regarded the PLA as possibly secondary to biliary infection (eTable 1 in Supplement 1). A PLA that was not likely or possibly secondary to biliary infection was considered as cryptogenic PLA.

The primary outcome was CRC diagnosis during the follow-up period, identified by administrative codes described in previous studies (eTable 1 in Supplement 1).^21,22,23^ We also collected mortality as a competing risk event. Mortality data were obtained from the VHA Vital Status File, which has excellent agreement with the National Death Index.^24^ Patients were followed up for up to 10 years from PLA diagnosis or until December 31, 2020. The *ICD-9* or *ICD-10* codes and *Current Procedural Terminology* codes were used to identify comorbidities and outcomes (eTable 1 in Supplement 1).

### Statistical Analysis

Data analysis was conducted from April 14, 2022, to October 31, 2023. Summary statistics are described using frequencies and percentages for categorical variables and means with SDs or medians with IQRs for continuous variables. The χ^2^ test was used to compare categorical variables, and the *t* test or Mann-Whitney test was used for continuous variables. A cumulative incidence function plot was used to describe CRC incidence over time while accounting for mortality as a competing event for patients with PLA and controls separately. Patients who were lost to follow-up or survived without CRC diagnosis for 10 years after PLA diagnosis or by December 31, 2020, were censored. Lost to follow-up was defined as no visit with primary care for 2 years. The Gray test was used to compare cumulative incidence curves between the 2 groups. A multivariable Fine-Gray subdistribution hazard regression model was fitted to assess time to CRC diagnosis between patients with PLA and controls while accounting for mortality as a competing event.^25,26^ We considered all demographic characteristics, comorbidities, and CRC screening within 5 years before PLA diagnosis (or corresponding index day for controls) as candidate variables for adjustments and selected for the final multivariable model with a backward elimination. In this analysis, we used a time-dependent coefficient approach by including an interaction term between PLA diagnosis and the logarithm of time (ie, log_10_ [days after PLA diagnosis +1]) to estimate the time-varying hazard ratio (HR) of CRC incidence over varying follow-up intervals.^27,28^ We chose to use this approach with the assumption that the association of PLA and CRC incidence does not remain constant over a 10-year period; thus, the consideration of nonproportional hazard was necessary. As post hoc subgroup analyses, we compared PLA and CRC among different races (White or Black), among patients with PLA likely from biliary infection, patients with PLA possibly from biliary infection, and patients with cryptogenic PLA. Race was included in the post hoc subgroup analysis because the incidence of colorectal cancer varies by race.

To assess whether preceding PLA had an association with the outcome of CRC, we also fitted a multivariable Cox regression model with time-dependent coefficient among patients who developed CRC during the follow-up period, including the presence or absence of preceding PLA diagnosis as a covariate. Multivariable Cox regression models were also adjusted for demographic characteristics and comorbidities using backward elimination to select variables for the final model. To assess whether there was an association between early CRC diagnosis and mortality, we also compared the hazard of mortality between patients with CRC diagnosed within 6 months and after 6 months from PLA using the multivariable Cox regression model. All inferential analyses were 2-tailed, with α<.05 being considered statistically significant. All analyses were conducted using SAS, version 9.4 (SAS Institute LLC).

## Results

In total, 8286 patients with PLA from 127 VHA hospitals were included. The positive predictive value of the diagnostic codes for PLA was 87% based on manual medical records review. The annual incidence rates of PLA increased from 5.38 cases per 100 000 patient-years in 2003-2005 to 9.02 cases per 100 000 patient-years in 2018-2020. We identified 23 201 patient-level matched controls without a diagnosis of PLA (Table 1). The mean (SD) age of patients with PLA was 65.8 (11.9) years and that of controls was 65.3 (11.7) years. Most patients were male (96.5% of PLA patients and 96.3% of controls). The most common race was White (68.6% of PLA patients and 71.5% of controls) followed by Black (18.8% of PLA patients and 15.2% of controls). Patients with PLA had more comorbidities compared with controls. Among patients with PLA, 1471 (17.8%) had a concurrent biliary infection diagnosis; therefore, the PLA was considered likely secondary to biliary infection. In addition, 1259 patients (15.2%) with PLA had a condition predisposing to biliary infection and were categorized as having PLA secondary to biliary infection. In contrast, PLAs in 6053 patients (73.1%) were considered cryptogenic.

**Table 1.** Baseline Characteristics of Patients With PLA and Matched Controls Without a Diagnosis of Infection

Characteristic	Patients with PLA (n = 8286), No. (%)	Matched cohort (n = 23 201), No. (%)	*P* value
Age, mean (SD)	65.8 (11.9)	65.3 (11.7)	.18
Sex			
Male	7994 (96.5)	22 347 (96.3)	.51
Female	292 (3.5)	854 (3.7)	
Race			
White	5682 (68.6)	16 592 (71.5)	<.001
Black	1557 (18.8)	3524 (15.2)	
Other^a^	1047 (12.6)	3085 (13.3)	
Moderate to high-risk alcohol consumption^b^	500 (6.0)	1483 (6.4)	.25
Social Vulnerability Index, No. of participants^c^	8097	22 701	
<0.25	1362 (16.8)	4105 (18.1)	.01
0.25 to <0.50	1880 (23.2)	5481 (24.1)	
0.50 to <0.75	2473 (30.5)	6894 (30.4)	
0.75 to 1.0	2382 (29.4)	6221 (27.4)	
Comorbidities, No. of participants	8121	23 575	
Myocardial infarction	692 (8.5)	1278 (5.5)	<.001
Congestive heart failure	1198 (14.8)	1901 (8.2)	<.001
COPD	2733 (33.7)	5890 (25.5)	<.001
PVD	1493 (18.4)	2877 (12.5)	<.001
CVD	1313 (16.2)	2692 (11.7)	<.001
Dementia	266 (3.3)	480 (2.1)	<.001
Hemiplegia	247 (3.0)	359 (1.6)	<.001
Autoimmune disease	292 (3.6)	620 (2.7)	<.001
Diabetes	3300 (40.6)	7481 (32.4)	<.001
Peptic ulcer	646 (8.0)	764 (3.3)	<.001
Liver disease	2467 (30.4)	1768 (7.7)	<.001
Chronic kidney disease	1241 (15.3)	1956 (8.5)	<.001
Solid cancer^d^	2539 (31.3)	3246 (14.1)	<.001
Hematologic cancer	235 (2.9)	332 (1.4)	<.001
HIV/AIDS	91 (1.1)	144 (0.6)	<.001
Prior colonoscopy/sigmoidoscopy within 5 y^e^	1886 (22.7)	4172 (18.0)	<.001
PLA likely from biliary infection	1471 (17.8)	NA	NA
PLA possibly from biliary infection	1259 (15.2)	NA	NA
Cryptogenic PLA	6053 (73.1)	NA	NA

Abbreviations: COPD, chronic obstructive pulmonary disease; CVD, cerebrovascular disease; NA, not applicable; PLA, pyogenic liver abscess; PVD, peripheral vascular disease.

^a^ Other includes Asian, Native American, Pacific Islander, multirace, unknown, declined to answer, or missing.

^b^ Defined as Alcohol Use Disorders Identification Test-C score greater than or equal to 5 (scale, 0-12; higher score indicates a greater likelihood drinking is affecting the patient’s health and safety).^19^

^c^ Defined using 2020 county-level database from the Centers for Disease Control and Prevention website.^20^

^d^ Excluded patients diagnosed with CRC before diagnosis of PLA (and before day of diagnosis in controls).

^e^ Colonoscopy or sigmoidoscopy conducted within the VHA system within 5 years before PLA diagnosis for PLA patients and index day for matched controls.

Diagnosis of CRC was found in a significantly higher proportion of patients with PLA compared with controls (1.9% [159 of 8286] vs 0.8% [196 of 23 201]; *P* < .001) (Table 2). Median days from PLA to CRC diagnosis was 147 (IQR, 32.5-681) days. In contrast, median days to CRC diagnosis from PLA diagnosis in the corresponding cases was 1000.5 (IQR, 405-1934) days among controls. The Figure shows the cumulative incidence function plot of CRC incidence over time among patients with PLA and controls. There was a large separation in CRC incidence between the 2 groups early after PLA diagnosis followed by parallel slopes. In the final multivariable Fine-Gray subdistribution hazard regression model, the CRC incidence was significantly higher among patients with PLA during the first 3 years after PLA diagnosis (HR, 3.64; 95% CI, 2.70-4.91 at 0.5 years; HR, 2.51; 95% CI, 1.93-3.26 at 1 year; HR, 1.74; 95% CI, 1.33-2.28 at 2 years; and HR, 1.41; 95% CI, 1.05-1.89 at 3 years) (Table 2; eTable 2 in Supplement 1).

**Table 2.** Time-Dependent HRs of Fine-Gray Subdistribution Regression Model to Estimate CRC Incidence

Variable	PLA, No./total No. (%)	Control, No./total No. (%)	*P* value	Time-dependent HR of CRC incidence, HR (95% CI)					
				0.5 y	1 y	2 y	3 y	4 y	5 y
Overall	159/8286 (1.9)	196/23 201 (0.8)	<.001	3.64 (2.70-4.91)	2.51 (1.93-3.26)	1.74 (1.33-2.28)	1.41 (1.05-1.89)	1.21 (0.88-1.67)	1.08 (0.76-1.52)
White	107/5682 (1.9)	138/15 876 (0.9)	<.001	3.72 (2.53-5.47)	2.59 (1.85-3.63)	1.81 (1.28-2.56)	1.47 (1.01-2.15)	1.27 (0.84-1.91)	1.13 (0.73-1.75)
Black	33/1557 (2.1)	32/4400 (0.7)	<.001	4.56 (1.90-10.93)	2.59 (1.20-5.55)	1.48 (0.70-3.13)	1.07 (0.49-2.34)	0.85 (0.37-1.94)	0.71 (0.30-1.70)
Likely secondary to biliary infection	16/1471 (1.1)	28/4054 (0.7)	.14	1.79 (0.77-4.13)	1.79 (0.86-3.74)	1.80 (0.85-3.79)	1.80 (0.81-4.01)	1.80 (0.76-4.25)	1.80 (0.72-4.49)
Possibly secondary to biliary infection	16/1259 (1.3)	37/3526 (1.1)	.52	1.50 (0.58-3.82)	1.31 (0.57-3.05)	1.16 (0.51-2.65)	1.08 (0.46-2.53)	1.02 (0.42-2.50)	0.98 (0.39-2.49)
Likely or possibly secondary to biliary infection	27/2233 (1.2)	48/6197 (0.8)	.06	1.78 (0.89-3.56)	1.64 (0.89-3.01)	1.52 (0.82-2.80)	1.45 (0.75-2.80)	1.40 (0.69-2.84)	1.37 (0.65-2.89)
Cryptogenic PLA	132/6053 (2.2)	148/17 004 (0.9)	<.001	4.27 (3.03-6.03)	2.77 (2.06-3.72)	1.81 (1.33-2.45)	1.41 (1.01-1.96)	1.18 (0.82-1.69)	0.92 (0.61-1.38)

Abbreviations: CRC, colorectal cancer; HR, hazard ratio; PLA, pyogenic liver abscess.

**Figure.**
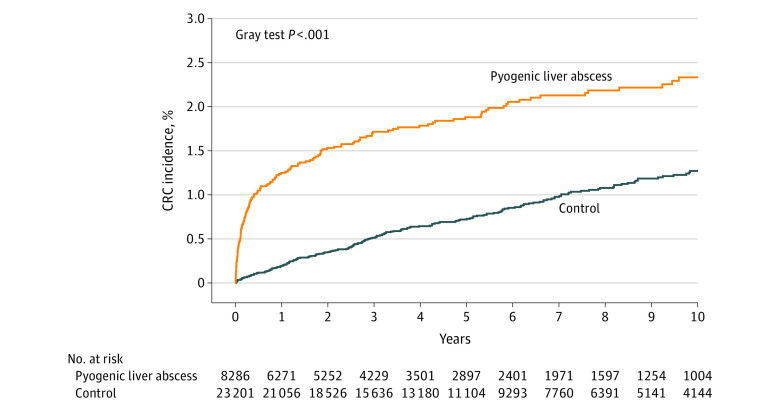
Cumulative Incidence Function Plots for Colorectal Cancer and for Patients With Pyogenic Liver Abscess and Controls Without a Diagnosis of Infection

In the post hoc subgroup analyses of Black and White patients, we observed that significantly higher proportions of patients with PLA were diagnosed with CRC during the follow-up period (Table 2; eFigure in Supplement 1). An association between PLA and CRC diagnosis was found in both racial groups in the Fine-Gray subdistribution regression model, although the difference was not significant at 3 years in Black patients, while White patients still had significantly increased HRs at that point (Table 2). This association was not observed among patients whose PLA was likely secondary to cholangitis or cholecystitis (HR, 1.78; 95% CI, 0.89-3.56 at 0.5 years) (Table 2; eFigure in Supplement 1). In contrast, there was a significant increase in CRC incidence among patients with cryptogenic PLA (Table 2; eFigure in Supplement 1).

A total of 2923 patients (35.3%) with PLA had positive culture results from blood or abscess (Table 3). Enterobacterales accounted for half of positive cultures (50.2%) followed by *Streptococcus* species (36.0%), anaerobic bacteria (22.6%), *Enterococcus* species (18.4%), and *Candida* species (7.9%). *Escherichia coli* was the most common bacteria among Enterobacterales (25.9%), followed by *Klebsiella* species (20.0%). We did not find any bacteria associated with a significantly increased CRC incidence.

**Table 3.** Profile of Microorganisms With CRC Incidence Among 2923 Patients Who Had Positive Cultures (Blood or Abscess) 14 Days Before and After PLA Diagnosis

Organism categories	No. (%)	
	Total	CRC incidence
Enterobacterales	1468 (50.2)	32 (2.2)
* Escherichia coli*	757 (25.9)	22(2.9)
*Klebsiella* species	585 (20.0)	10 (1.7)
* Klebsiella pneumoniae*	426 (14.6)	9 (2.1)
*Streptococcus* species	1052 (36.0)	24 (2.3)
*Streptococcus anginosus* group	409 (14.0)	12(2.9)
Anaerobic bacteria	661 (22.6)	20 (3.0)
*Enterococcus* species	537 (18.4)	8/(1.5)
*Candida* species	232 (7.9)	8 (3.4)
Non-fermentative gram-negative rods	178 (6.1)	4 (2.2)
Total	2923 (100)	64 (2.2)

Abbreviations: CRC, colorectal cancer; PLA, pyogenic liver abscess.

Among 355 patients (159 patients with PLA and 196 controls) who were diagnosed with CRC during the follow-up period, the hazards of mortality were significantly higher among patients with PLA up to 3 years from CRC diagnosis after adjusting for age and comorbidities (Table 4; eTable 3, eFigure in Supplement 1). Hazards of mortality among patients with CRC diagnosed within 6 months from PLA diagnosis were higher than those among patients with CRC diagnosed after 6 months from PLA diagnosis among 159 patients with PLA and CRC, although the difference was not statistically significant due to a wide CI (Table 4).

**Table 4.** Time-Dependent HRs of Cox Regression Model to Estimate the Mortality After CRC Diagnosis

Variable	Group, No. (%)	*P* value	Time-dependent HR of mortality (95% CI)					
			0.5 y	1 y	2 y	3 y	4 y	5 y
PLA vs control	PLA, 97 of 159 (61.0); control, 81 of 196 (41.3)	<.001	1.76 (1.26-2.47)	1.64 (1.20-2.26)	1.54 (1.09-2.17)	1.48 (1.01-2.16)	1.44 (0.95-2.17)	1.41 (0.91-2.18)
CRC within 6 mo after PLA vs CRC after 6 mo from PLA	Within 6 mo, 36 of 62 (58.1); after 6 mo, 39 of 97 (40.2)	.03	16.36 (0.91-294.95)	18.68 (0.86-404.28)	21.28 (0.82-551.56)	22.97 (0.80-661.27)	24.24 (0.78-752.21)	25.28 (0.77-831.32)

Abbreviations: CRC, colorectal cancer; HR, hazard ratio; PLA, pyogenic liver abscess.

## Discussion

In our patient-level matched retrospective cohort study among 127 VHA hospitals over an 18-year-period with 8286 patients with PLA, we found that the incidence of CRC was significantly higher among patients diagnosed with PLA compared with controls. Time-dependent hazard for CRC incidence was significantly higher among patients with PLA within 3 years after PLA diagnosis, but not significant after 3 years. The association between PLA and CRC incidence was observed among Black patients, White patients, and patients with cryptogenic PLA, but not among patients with PLA likely or possibly from biliary infection. The hazard for mortality was significantly higher among patients with CRC found after PLA compared with controls who developed CRC, especially if CRC was diagnosed within 6 months from PLA diagnosis. Our results showed higher HRs between cryptogenic PLA and incidence of CRC.

Patients with PLA had the highest HR for CRC incidence early after PLA diagnosis, which remained significantly high up to 3 years. Considering that CRC may disrupt the colonic mucosal barrier and allow colonic bacteria to invade the portal vein system, our findings suggest that undiagnosed CRC was the likely cause of cryptogenic PLA. The difference in the hazard of CRC incidence became less obvious after 3 years. This is discordant with the population-based study conducted in Taiwan, which reported that the cumulative incidence of CRC seemed constantly higher in patients with PLA during a 10-year period.^14^ The reasons for the difference in long-term risk for CRC remain uncertain, although the difference in population characteristics may be partly responsible. In addition, that population-based study as well as other studies^14,15,16^ might have affected the practice pattern of CRC screening after PLA. In our study, patients whose PLA was likely or possibly secondary to biliary infection did not have an increased CRC incidence rate compared with controls. This suggests that the association between PLA and CRC is likely to apply only among patients with cryptogenic PLA, which supports our hypothesis of the association between development of PLA and disrupted colonic mucosal barrier due to CRC.

We observed a higher HR of CRC incidence after PLA diagnosis between Black and White patients; however, the difference in the hazard of CRC was not significant after 3 years among Black patients. This difference may be partly explained by the smaller number of patients and wider CIs in the Black patient subgroup. For bacteria identified from blood or abscess cultures, we did not find any particular microorganisms associated with CRC incidence. This is discordant with previous small observational studies that suggested an association between PLA caused by *Klebsiella pneumoniae* and CRC incidence.^29,30,31^ However, the number of CRCs that occurred in the presence of each microorganism was limited. Therefore, our study might have been underpowered to investigate the link between CRC incidence and specific microorganisms.

The Cox regression model adjusted for demographic characteristics and comorbidities suggests that the hazard for mortality was significantly higher among patients with CRC after PLA compared with patients with CRC among controls. We are unaware of any previous study that compared the outcome of CRC among patients with PLA and controls. We suspect that CRCs after PLA would have rapidly progressed or been at an advanced stage at the time of PLA diagnosis to cause mucosal barrier disruption compared with CRC occurring in controls, although we cannot estimate this without detailed information. Among those with CRC after PLA diagnosis, the hazard for mortality was higher in patients whose CRC was diagnosed within 6 months likely due to an advanced stage in the early diagnosis group, which prompted more immediate workup. Considering the worse outcome of CRC associated with PLA, clinicians should be aware of the association between cryptogenic PLA and CRC.

### Strengths and Limitations

We believe our study is the largest outside Southeast Asia to assess whether PLA is associated with CRC. Our study results are largely concordant with a population-based study conducted in Taiwan,^14^ except for the increased HR for CRC incidence not observed after 3 years from PLA diagnosis. The incidence rate of PLA was increasing in our study, which is in line with results from a population-based study of PLA in the US that reported a 4.1% annual increase in incidence rates of PLA from 1994 to 2005.^32^ The association between PLA and CRC deserves further study given the increasing incidence of PLA in the US diagnosis of serious illness, including PLA, is an opportunity for patients to consider CRC screening, even for those not previously interested, given the suboptimal CRC screening rates in many settings despite the introduction of new screening modalities.^33^

There are several limitations to our study. First, we were not able to control for important risk factors, such as smoking or family history of CRC, due to the inability to capture those factors from the database. Inability to control for those variables in the multivariable model might have affected our results. Second, our study was conducted using administrative codes to identify variables. We used administrative codes that were used in previous studies,^14,21,22,23,32^ and our manual medical records review of 100 randomly selected patients verified the accuracy of diagnostic codes for PLA, but it is still possible some cases were misclassified. Other conditions were also not captured without medical records review. For example, we were not able to identify routes of PLA other than biliary infection, such as bacteremia from systemic infection, direct extension from a contiguous focus of infection, or penetrating trauma, which would ideally have been classified separately. Similarly, we were not able to capture CRC characteristics, such as pathologic test results or stage at time of diagnosis, which limited our analyses interpretation for mortality after CRC diagnosis. Third, we were unable to capture CRC diagnoses made outside the VHA system. We believe we captured most CRC cases because most of the participants received care, including preventive medicine, within the VHA system. Fourth, we were not able to capture colonoscopy or sigmoidoscopy conducted outside the VHA system, as well as other procedures related to CRC screening or diagnosis, such as fecal occult blood testing. Thus, we were unable to further investigate the possible association between PLA and CRC incidence stratified by CRC screening status. Fifth, our study was conducted using the VHA cohort whose participants were mainly older men. It is not clear how our results apply to the non-VHA population, especially women.

## Conclusions

In this cohort study, patients who were diagnosed with cryptogenic PLA had a significantly higher incidence of CRC after 3 years from PLA diagnosis. These findings suggest that offering CRC screening to patients with cryptogenic PLA may be useful, especially in patients who have not been screened according to guidelines.

## Notes

Supplement 1. **eTable 1.** Definitions of Variables **eTable 2.** Final Multivariate Subdistribution Regression Model to Estimate CRC Incidence Accounting for Mortality as a Competing Risk **eTable 3.** Final Multivariate Cox Regression Model to Estimate Mortality After CRC Diagnosis **Figure.** Cumulative Incidence Function Plots for CRC Incidence Rates Among Post Hoc Subgroup Analyses

Supplement 2.
Data Sharing Statement
