# Supplementary material for: Incidence of Colorectal Cancer in Patients Diagnosed With Pyogenic Liver Abscess
Source: JAMA Netw Open. 2023 Dec 18;6(12):e2348218. doi: 10.1001/jamanetworkopen.2023.48218 (PMC10728768; doi:10.1001/jamanetworkopen.2023.48218)
Supplement: Supplement 1. — eTable 1. Definitions of Variables eTable 2. Final Multivariate Subdistribution Regression Model to Estimate CRC Incidence Accounting for Mortality as a Competing Risk eTable 3. Final Multivariate Cox Regression Model to Estimate Mortality After CRC Diagnosis Figure. Cumulative Incidence Function Plots for CRC Incidence Rates Among Post Hoc Subgroup Analyses [file jamanetwopen-e2348218-s001.pdf]

Supplementary Online Content

Suzuki H, Kidder I, Tanaka T, Goto M. Incidence of colorectal cancer in patients diagnosed with pyogenic liver abscess. *JAMA Netw Open*. 2023;6(12):e2348218. doi:10.1001/jamanetworkopen.2023.48218

eTable 1. Definitions of Variables

eTable 2. Final Multivariate Subdistribution Regression Model to Estimate CRC Incidence Accounting for Mortality as a Competing Risk

eTable 3. Final Multivariate Cox Regression Model to Estimate Mortality After CRC Diagnosis

Figure. Cumulative Incidence Function Plots for CRC Incidence Rates Among Post Hoc Subgroup Analyses

This supplementary material has been provided by the authors to give readers additional information about their work.

© 2023 Suzuki H et al. *JAMA Network Open.*

eTable 1. Definitions of variables.

Variable	International Classification of Diseases (ICD)-9 or -10 Codes and Current Procedural Terminology (CPT) Codes
Pyogenic liver abscess	ICD-9: 572.0<br>ICD-10: K75.0
Colorectal cancer	ICD-9: 153, 154.0, 154.1<br>ICD-10: C18, C19, C20
Biliary infections which defined patients with PLA likely from a biliary infection	(From 28 days before to 7 days after PLA diagnosis)<br>ICD-9: 574.0, 574.1, 574.3, 574.4, 574.6, 574.7, 574.8, 575.0, 575.1, 575.4, 575.5, 576.1, 576.2, 576.3, 576.4<br>ICD-10: K80.0, K80.1, K80.3, K80.4, K80.6, K81, K82.2, K82.3, K82.A, K83.0, K83.1
Conditions predisposing to biliary infection which defined patients with PLA possibly from biliary infection	(Before PLA diagnosis)<br>Previous common bile duct surgery<br>ICD-9: 51, ICD-10: 0F19<br>Previous ERCP with sphincterotomy<br>CPT: 43261, 43262, 43264, 43265, 43273, 43274, 43275, 43277<br>Liver transplant status<br>ICD-9: 996.82, V42.7 ICD-10: T86.40, Z94.4<br>Cancer of biliary tract or pancreatic head<br>ICD-9: 156, 157.0, ICD-10: C24, C25.0<br>Cholelithiasis without infection<br>ICD-9: 574.2, 574.5, 574.9, ICD-10: K80.2, K80.5, K80.7, K80.8
Myocardial infarction	ICD-9: 410, 412<br>ICD-10: I21, I22, I25.2
Congestive heart failure	ICD-9: 298.91, 402.01, 402.11, 402.91, 404.91, 404.01, 404.03, 404.11, 404.13, 404.91, 404.93, 428<br>ICD-10; I09.9, I11.0, I13.0, I13.2, I25.5, I42.0, I42.5, I42.6, I42.7, I42.8, I42.9, I43, I50, P29.0
Peripheral vascular disease	ICD-9: 440, 441, 443.1, 443.2, 443.3, 443.4, 443.5, 443.6, 443.7, 443.8, 443.9, 447.1, 557.1, 557.9, V43.4, 093.0, 437.3<br>ICD-10: I70, I71, I73.1, I73.8, I73.9, I77.1, I79.0, I79.2, K55.1, K55.8, K55.9, Z95.8, Z95.9
Cerebrovascular disease	ICD-9: 362.34, 430, 431, 432, 433, 434, 435, 436, 437, 438<br>ICD-10: G45, G46, I60, I61, I62, I63, I64, I65, I66, I67, I68, I69, H34.0
Dementia	ICD-9: 290, 294.1, 331.2<br>ICD-10: F00, F01, F02, F03, F05.1 G30, G31.1
COPD	ICD-9: 416.8, 416.9, 490-505, 506.4, 508.1, 508.8<br>ICD-10: I27.8, I27.9, J40-47, J60-67, J68.4, J70.1, J70.3
Autoimmune disease	ICD-9: 446.5, 710.0, 710.1, 710.2, 710.3, 710.4, 714.0, 714.1, 714.2, 714.8, 725<br>ICD-10: M05, M06, M31.5, M32, M33, M34, M35.1, M35.3, M36.0
Peptic ulcer	ICD-9: 531-534<br>ICD-10: K25-28

© 2023 Suzuki H et al. *JAMA Network Open.*

Liver disease	Mild<br>ICD-9: 070.22, 070.23, 070.32, 070.33, 070.44, 070.54, 070.6, 070.9, 570, 571, 573.3, 573.4, 573.8, 573.9, V42.7<br>ICD-10: B18, K70.0, K70.1, K70.2, K70.3, K70.9, K71.3, K71.4, K71.5, K71.7, K73, K74, K76.0, K76.2, K76.3, K76.4, K76.8, K76.9, Z94.4<br>Moderate to severe<br>ICD-9: 456.0, 456.1, 456.2, 572.2, 572.3, 572.4, 572.5, 572.6, 572.7, 572.8<br>ICD-10: I85.0, I85.9, I86.4, I98.2, K70.4, K71.1, K72.1, K72.9, K76.5, K76.6, K76.7
Diabetes mellitus	No complication<br>ICD-9: 250.0, 250.1, 250.2, 250.3, 250.8, 250.9<br>ICD-10: E10.0, E10.1, E10.6, E10.8, E10.9, E11.0, E11.1, E11.6, E11.8, E11.9, E12.0, E12.1, E12.6, E12.8, E12.9, E13.0, E13.1, E13.6, E13.8, E13.9, E14.0, E14.1, E14.6, E14.8, E14.9<br>With complication<br>ICD-9: 250.4, 250.5, 250.6, 250.7<br>ICD-10: E10.2, E10.3, E10.4, E10.5, E10.7, E11.2, E11.3, E11.4, E11.5, E11.7, E12.2, E12.3, E12.4, E12.5, E12.7, E13.2, E13.3, E13.4, E13.5, E13.7, E14.2, E14.3, E14.4, E14.5, E14.7
Hemiplegia	ICD-9: 334.1, 342, 343, 344.0, 344.1, 344.2, 344.3, 344.4, 344.5, 344.6, 344.9<br>ICD-10: G04.1, G11.4, G80.1, G80.2, G81, G82, G83.0, G83.1, G83.2, G83.3, G83.4, G83.9
Renal disease	ICD-9: 403.01, 403.11, 403.91, 404.02, 404.03, 404.12, 404.13, 404.92, 404.93, 582, 583.0, 583.1, 583.2, 583.3, 583.4, 583.5, 583.6, 583.7, 585, 586, 588.0, V42.0, V45.1, V56<br>ICD-10: I12.0, I13.1, N03.2, N03.3, N03.4, N03.5, N03.6, N03.7, N05.2, N05.3, N05.4, N05.5, N05.6, N05.7, N18, N19, N25.0, Z49.0, Z49.1, Z49.2, Z94.0, Z94.2
Solid cancer other than colorectal cancer	No metastasis<br>ICD-9: 140-172, 174.0, 195.8, 200-208<br>ICD-10: C00-26, C30-34, C37-41, C43, C45-58, C60-76<br>Metastatic<br>ICD-9: 196-199<br>ICD-10: C77-80<br>(Excluding colorectal cancer<br>ICD-9: 153, 154.0, 154.1<br>ICD-10: C18, C19, C20)
Hematologic malignancy	Lymphoma<br>ICD-9: 200, 201, 202, 203.0, 238.6<br>ICD-10: C81-88<br>Leukemia<br>ICD-9: 204, 208<br>ICD-10: C91-95
HIV/AIDS	ICD-9: 042-044<br>ICD-10: B20, B21, B22, B24
Colonoscopy	ICD-9: V76.51

© 2023 Suzuki H et al. *JAMA Network Open.*

	ICD-9PS: 45.23<br>ICD-10: Z12.11<br>ICD-10PS: 0DJD8ZZ, 0DBE8ZX, 0DBF8ZX, 0DBG8ZX, 0DBH8ZX, 0DBK8ZX, 0DBL8ZX, 0DBM8ZX, 0DBN8ZX<br>CPT: 44388, 44389, 44390, 44391, 44392, 44393, 44394, 44397, 44401, 44402, 44403, 4404, 44405, 44406, 44407, 44408, 45378, 45379, 45380, 45381, 45382, 45383, 45384, 45385, 45386, 45387, 45388, 45389, 45390, 45391, 45392, 45393, 45398, 45535, G0105, G0121, G6019, G6020, G6024, G6025
Sigmoidoscopy	ICD-9PS: 45.24<br>ICD-10PS: 0DJD8ZZ<br>CPT: 45300, 45303, 45305, 45307, 45308, 45309, 45315, 45317, 45320, 45321, 45327, 45330, 45331, 45332, 45333, 45334, 45335, 45337, 45338, 45339, 45340, 45341, 45342, 45345, 45346, 45347, 45349, 45350, G0104, G6022, G6023

PLA: pyogenic liver abscess, CRC: colorectal cancer, COPD: chronic obstructive pulmonary disease; HIV: human immunodeficiency virus; AIDS: acquired immunodeficiency syndrome

© 2023 Suzuki H et al. *JAMA Network Open.*

eTable 2. Final multivariate subdistribution regression model to estimate CRC incidence accounting for mortality as a competing risk.

Characteristic	Hazard ratio (95% CI)
Liver abscess	56.14 (20.00-157.54)
Time (days)	0.59 (0.50-0.70)
Age	
<45	Reference
45-54	3.36 (1.00-11.28)
55-64	5.86 (1.86-18.49)
65-74	6.07 (1.93-19.13)
75-84	6.94 (2.18-22.15)
≥85	3.86 (1.08-13.86)
Race	
White	Reference
Black	1.55 (1.18-2.03)
Other	1.00 (0.71-1.42)
Moderate to high-risk alcohol consumption	1.50 (1.03-2.19)
Comorbidities and others	
Liver disease	0.68 (0.48-0.97)
Autoimmune disease	1.59 (1.18-2.03)
Solid cancer	1.05 (0.80-1.39)
Prior colonoscopy/sigmoidoscopy within 5 years	0.65 (0.50-0.85)

CRC: colorectal cancer; COPD: chronic obstructive pulmonary disease

All demographics, comorbidities, and CRC screening within five years before PLA diagnosis (or corresponding index day for controls) were used as candidate variables for adjustments and selected for the final multivariable model with a backward elimination using >0.1 as criteria for removal.

© 2023 Suzuki H et al. *JAMA Network Open.*

eTable 3. Final multivariate Cox regression model to estimate mortality after CRC diagnosis.

Characteristic	Hazard ratio (95% CI)
Liver abscess	2.93 (0.99-8.73)
Time (days)	0.91 (0.76-1.09)
Age	
<45	Reference
45-54	4.46 (0.55-36.29)
55-64	3.14 (0.41-24.00)
65-74	2.99 (0.39-22.77)
75-84	3.08 (0.41-23.38)
>=85	8.34 (1.02-68.33)
Comorbidities	
Myocardial infarction	0.40 (0.23-0.68)
COPD	0.73 (0.51-1.03)
Peripheral vascular disease	1.62 (1.02-2.57)
Diabetes	1.52 (1.11-2.10)
Renal disease	0.39 (0.22-0.69)
Solid cancer other than colorectal cancer	1.62 (1.14-2.31)
Hematologic malignancy	0.26 (0.10-0.64)

CRC: colorectal cancer; COPD: chronic obstructive pulmonary disease

All demographics and comorbidities were used as candidate variables for adjustments and selected for the final multivariable model with a backward elimination using >0.1 as criteria for removal.

© 2023 Suzuki H et al. *JAMA Network Open.*

Figure. Cumulative incidence function plots for CRC incidence rates among post hoc subgroup analyses.

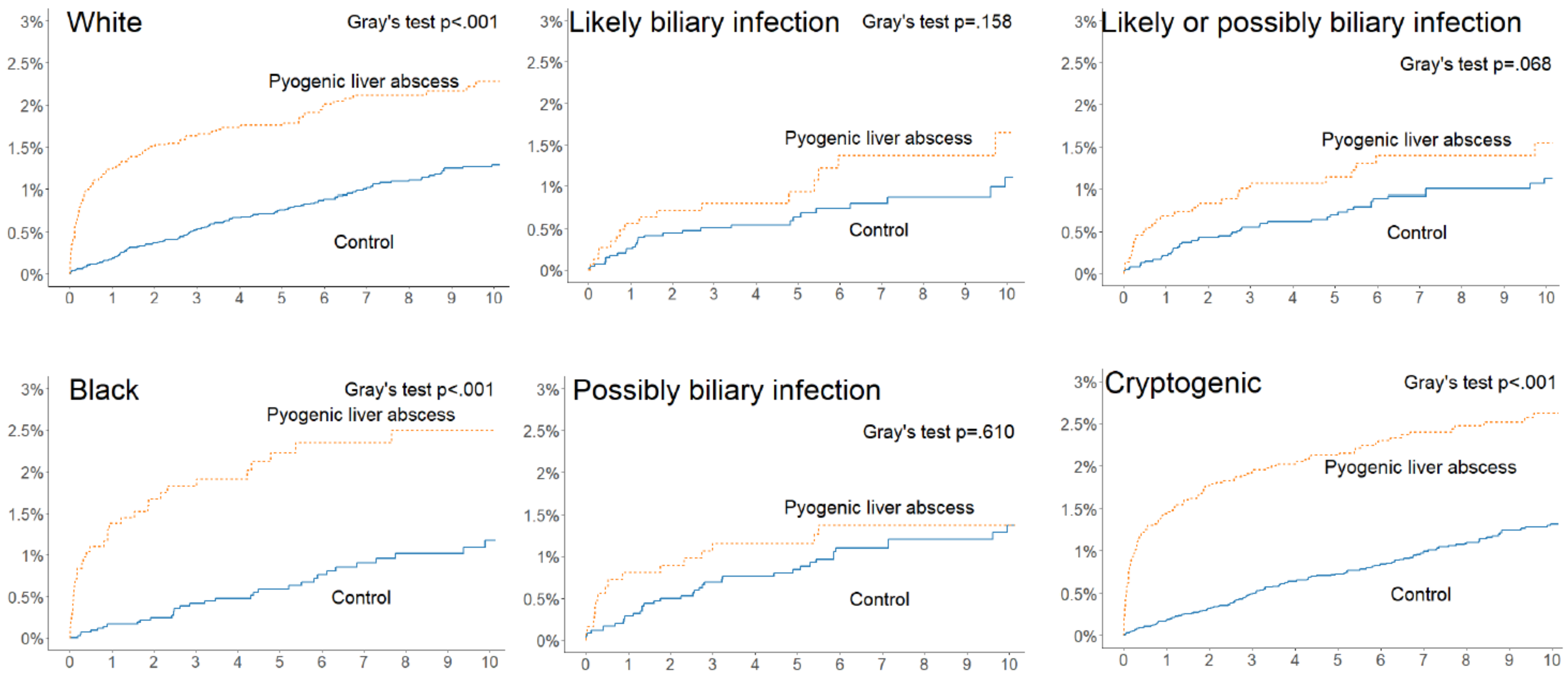

X-axis: Years from pyogenic liver abscess diagnosis, Y-axis: Proportion of patients who developed colorectal cancer

© 2023 Suzuki H et al. *JAMA Network Open.*
