# Supplementary material for: Incidence of Colorectal Cancer in Patients Diagnosed With Pyogenic Liver Abscess
Source: JAMA Netw Open. 2023 Dec 18;6(12):e2348218. doi: 10.1001/jamanetworkopen.2023.48218 (PMC10728768; doi:10.1001/jamanetworkopen.2023.48218)
Supplement: Supplement 2. — Data Sharing Statement [file jamanetwopen-e2348218-s002.pdf]

Data Sharing Statement

Suzuki. Incidence of Colorectal Cancer in Patients Diagnosed With Pyogenic Liver Abscess. *JAMA Netw Open*. Published December 18, 2023. doi:10.1001/jamanetworkopen.2023.48218

Data
Data available: No

Additional Information
Explanation for why data not available: Using VHA data which is not publicly available
